# Elastic sowing dates with low seeding rate for grain yield maintenance in mechanized large-scale double-cropped rice production

**DOI:** 10.1038/s41598-020-66175-7

**Published:** 2020-06-08

**Authors:** Huabin Zheng, Bo Li, Yuanwei Chen, Qiyuan Tang

**Affiliations:** College of Agronomy, Hunan Agricultural University, Changsha, 410128 P.R. China

**Keywords:** Plant sciences, Environmental impact

## Abstract

Elastic sowing dates (ESDs) are correlated with rice grain yield. ESD is the easiest factor for farmers to manipulate in mechanized large-scale farming. In this study, field experiments were conducted over a 2-year period to determine the effects of different sowing dates on growth duration, effective accumulated temperature, and yield attributes in two early- and late-season machine-transplanted rice cultivars. In early rice (ER), a delay in the sowing date led to decreased grain yield and shorter growth duration. In late rice (LR), delayed sowing led to significantly lower grain yield and prolonged growth duration. In LR, significantly positive correlations were detected between effective accumulated temperature in the post-heading stage and both filling ratio and yield. Reproductive redundancy increased markedly in LR, by 7.72% over a 5-day interval. We determined that the ESDs for LR were 10 days later than the control, and that of ER was recommend early sowing rather than late sowing. These findings suggest a new strategy to meet the demands of mechanized large-scale rice farming: the development of thermal sensitive high-yield long-duration ER cultivars and high-yield short-duration LR cultivars.

## Introduction

Double rice cropping is a typical rice production system in China, accounting for >20% of the total rice production area, and is considered an efficient system for the improvement of multiple-crop indices and total rice production^1^. Yang *et al*.^2^ reported that rice production in China increased by 4.0% with the concomitant favorable adoption of a multiple-cropping system. Therefore, rice production plays a pivotal role in ensuring national food security^3^. Machine transplanting is a labor-saving alternative rice cultivation technology^4^. As efficient agriculture and land transfer systems have been popularized in recent years, mechanized large-scale farming technology has developed rapidly for rice production in China^5,6^, leading to increases in the time required for farming operations (e.g., land preparation and seedling transplantation)^7^; therefore, reasonable seedling transplantation practices in terms of sowing date and seedling age are needed. Important non-monetary factors affecting potential rice yield include transplanting time (sowing date if seedling age is constant) and seedling age at transplanting^8^. Sowing date is correlated to rice grain yield, and is the easiest factor for producers to manipulate^9,10^. However, little information is available on the role of elastic sowing dates (ESDs) in mechanized large-scale farming because seedling age is generally limited to 20 days for early rice (ER) and 15 days for late rice (LR).

Under small-scale manual transplanting practices, ESDs are March 25–30 for ER and June 25–28 for LR in China. Due to increases in the length of farming operations in mechanized large-scale farming systems, delayed sowing dates have been adopted by most farmers; however, delayed sowing reduces the effectiveness of accumulated temperature due to decreasing daily temperatures in LR fields^6^, resulting in poor rice growth and development, and ultimately grain yield loss. Furthermore, ESDs for ER necessarily affect those for LR. Therefore, ESDs for ER and LR should be improved. In this study, we investigated ESDs for ER and LR to maintain yield in mechanized large-scale farming systems. We conducted field experiments over a 2-year study period to determine the effects of ESDs on growth duration, effective accumulated temperature, and yield attributes of ER and LR cultivars under machine-transplanted conditions.

## Results

### Growth duration vary with different sowing dates

Growth duration was shorter for ER with delayed sowing (Table 1). A 15-day delay in sowing led to a 3-day reduction in growth duration compared with the control. In LR, growth duration was prolonged as sowing date was delayed; the growth duration following a 20-day delay in sowing was more than 8 days longer than that of the control, especially in the reproductive stage. Total effective accumulated temperature exhibited the opposite trend. Growth duration decreased and significantly increased as effective accumulated temperature increased in ER and LR, respectively (Table 2). Effective accumulated temperature decreased by an average of 52.2 °C in LR, with a 5-day interval.

**Table 1:** Growth duration in machine-transplanted double-cropped rice grown under different sowing periods in 2015 and 2016.

Season	Cultivar	Sowing date delay (days)	FH (date)	MA (date)	Days from TP to FH	Days from FH to MA	Growth duration (days)
2015 ER	ZZ39	+ 0	6–10	7–10	54	30	105
		+5	6–14	7–14	52	30	104
		+10	6–19	7–20	53	31	104
		+15	6–23	7–22	52	29	102
2016 ER	ZZ39	+ 0	6–16	7–12	63	26	109
		+5	6–18	7–16	61	27	108
		+10	6–20	7–20	59	28	107
		+15	6–23	7–23	55	30	105
	LLY268	+ 0	6–21	7–16	68	25	113
		+5	6–23	7–20	65	27	112
		+10	6–26	7–25	64	28	112
		+15	6–29	7–28	61	29	110
	Average	+0			62 ± 7a	27 ± 3a	109 ± 4a
		+5			59 ± 7a	28 ± 2a	108 ± 4a
		+10			59 ± 6a	29 ± 2a	108 ± 4a
		+15			56 ± 5a	29 ± 1a	106 ± 4a
		LSD0.05			11	3	8
2015 LR	HY518	+ 0	9–10	10–13	53	33	110
		+5	9–12	10–22	55	40	114
		+10	9–17	10–26	54	39	113
		+15	9–20	11–02	52	43	115
		+20	10–2	11–13	59	42	121
2016 LR	HY518	+ 0	9–09	10–18	55	40	109
		+5	9–14	10–23	56	39	109
		+10	9–18	10–28	55	40	109
		+15	9–22	11–03	54	42	110
		+20	9–28	11–10	56	42	112
	TY390	+ 0	9–14	10–25	60	42	116
		+5	9–18	11–02	60	45	119
		+10	9–22	11–09	59	48	121
		+15	9–28	11–16	61	48	123
		+20	10–4	11–22	62	50	126
	Average	+0			56 ± 4a	38 ± 5a	112 ± 4a
		+5			57 ± 3a	41 ± 3a	114 ± 5a
		+10			56 ± 3a	42 ± 5a	114 ± 6a
		+15			56 ± 5a	44 ± 3a	116 ± 7a
		+20			59 ± 3a	45 ± 5a	120 ± 7a
		LSD0.05			6	8	11

TP, transplanting stage; FH, full heading stage; MA, mature stage. LSD, least significant difference at a significance level of *P* < 0.05.

**Table 2:** Effective accumulated temperature in machine-transplanted double-cropped rice grown under different sowing periods in 2015 and 2016.

Season	Cultivar	Sowing date delay (days)	Effective accumulated temperature (°C)			
			SO to TP	TP to FH	FH to MA	Total
2015 ER	ZZ39	+0	126	673	475	1274
		+5	117	670	493	1281
		+10	136	699	506	1341
		+15	197	692	475	1364
2016 ER	ZZ39	+ 0	145	749	457	1351
		+5	154	763	476	1392
		+10	144	781	487	1412
		+15	145	751	533	1429
	LLY268	+ 0	145	847	434	1425
		+5	154	846	468	1468
		+10	144	846	524	1514
		+15	145	836	560	1540
		+0	139 ± 11a	756 ± 88a	455 ± 21b	1350 ± 76a
		+5	142 ± 21a	760 ± 88a	479 ± 13ab	1380 ± 94a
		+10	141 ± 5a	775 ± 74a	506 ± 19a	1422 ± 87a
		+15	162 ± 30a	760 ± 72a	523 ± 43a	1444 ± 89a
		LSD0.05	36	152	50	163
2015 LR	HY518	+ 0	336	985	376	1697
		+5	307	1033	448	1788
		+10	314	915	430	1659
		+15	343	839	423	1605
		+20	351	887	316	1553
2016 LR	HY518	+ 0	272	1039	500	1811
		+5	287	1035	467	1789
		+10	300	985	432	1718
		+15	319	925	395	1639
		+20	323	925	340	1588
	TY390	+ 0	272	1091	482	1845
		+5	287	1095	444	1826
		+10	300	1037	423	1761
		+15	319	1018	364	1701
		+20	323	992	357	1671
		+0	293 ± 37b	1038 ± 53ab	453 ± 67a	1784 ± 78a
		+5	294 ± 12b	1054 ± 35a	453 ± 12a	1810 ± 22a
		+10	305 ± 8ab	979 ± 61ab	428 ± 5a	1713 ± 51ab
		+15	327 ± 14ab	927 ± 90b	394 ± 30ab	1648 ± 49bc
		+20	332 ± 16a	935 ± 53b	338 ± 21b	1604 ± 61c
		LSD0.05	37	111	63	100

SO, sowing stage; TP, transplanting stage; FH, full heading stage; MA, mature stage. LSD, least significant difference at a significance level of *P* < 0.05.

### Reproductive redundancy and nutrition redundancy under different sowing dates

**Figure 1:**
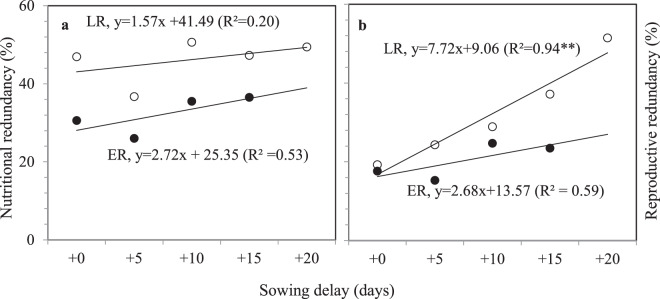
Variation in nutritional and reproductive redundancy (%) in machine-transplanted double-cropped rice with delayed sowing in 2015 and 2016.

No significant difference in aboveground biomass was observed in ER or LR among different sowing dates (Table 3); aboveground biomass was 1467–1526 g m^−2^ in LR. Variation in crop growth rate (CGR) exhibited a similar trend. Harvest index (HI) declined in both ER and LR as sowing date was postponed (Table 3), and decreased dramatically in LR, by 3.8% with a 5-day interval. Reproductive redundancy increased by 2.68 and 7.72% in ER and LR, respectively, with a 5-day interval, following sowing date delay (Fig. 1b). Nutrition redundancy increased by 2.72 and 1.57% in ER and LR, respectively, with a 5-day interval following sowing date delay (Fig. 1a).

**Table 3:** Aboveground biomass, crop growth rate (CGR), and harvest index (HI) in machine-transplanted double-cropped rice under different sowing periods in 2015 and 2016.

Season	Cultivar	Sowing date delay (days)	Aboveground biomass (g m^−2^)		CGR		HI
			FH	MA	FH	MA	
2015 ER	ZZ39	+0		954		11.0	63.5
		+5		979		11.7	62.7
		+10		1158		14.0	58.2
		+15		1042		12.6	57.0
2016 ER	ZZ39	+ 0	703	1330	11.2	12.2	59.2
		+5	771	1348	12.6	12.5	60.3
		+10	639	1174	10.8	11.0	56.2
		+15	636	1291	11.6	12.3	57.4
	LLY268	+ 0	863	1333	12.4	11.8	53.7
		+5	756	1363	11.6	12.2	52.5
		+10	801	1332	12.5	11.9	43.2
		+15	847	1383	13.9	12.6	46.3
		+0	783 ± 113a	1206 ± 218a	11.8 ± 0.8a	11.7 ± 0.6a	58.8 ± 4.9a
		+5	764 ± 11a	1230 ± 218a	12.1 ± 0.7a	12.1 ± 0.4a	58.5 ± 5.3a
		+10	720 ± 115a	1221 ± 96a	11.7 ± 1.2a	12.3 ± 1.5a	52.5 ± 8.1a
		+15	742 ± 149a	1239 ± 176a	12.8 ± 1.6a	12.5 ± 0.2a	53.6 ± 6.3a
		LSD0.05	305	346	3.2	1.6	11.9
2015 LR	HY518	+ 0		1216		14.1	59.7
		+5		1116		11.7	51.8
		+10		1102		11.8	56.6
		+15		1231		13.0	54.0
		+20		1013		10.0	46.9
2016 LR	HY518	+ 0	957	1825	17.1	19.2	54.4
		+5	895	1721	16.0	18.1	53.1
		+10	1010	1713	18.4	18.0	53.0
		+15	1046	1761	19.4	18.3	48.0
		+20	989	1712	18.0	17.8	41.7
	TY390	+ 0	975	1538	16.0	17.3	53.2
		+5	1116	1868	18.6	17.8	48.8
		+10	1058	1632	17.9	15.2	47.6
		+15	944	1678	15.7	15.5	42.0
		+20	1117	1676	18.3	15.8	26.4
		+0	966 ± 13a	1526 ± 305a	16.6 ± 0.8a	16.9 ± 2.6a	55.8 ± 3.5a
		+5	1006 ± 156a	1568 ± 399a	17.3 ± 1.8a	15.9 ± 3.6a	51.2 ± 2.2a
		+10	1034 ± 34a	1482 ± 332a	18.2 ± 0.4a	15.0 ± 3.1a	52.4 ± 4.5a
		+15	995 ± 72a	1557 ± 285a	17.6 ± 2.6a	15.6 ± 2.7a	48.0 ± 6.0ab
		+20	1053 ± 91a	1467 ± 394a	18.2 ± 0.2a	14.5 ± 4.1a	38.3 ± 10.7b
		LSD0.05	227	629	3.8	5.9	11.1

FH, full heading stage; MA, mature stage. LSD, least significant difference at *P* < 0.05.

### Yield difference under different sowing dates

Grain yield in ER and LR decreased as sowing date delay increased (Table 4). In LR, grain yields differed significantly among different sowing dates (*P* < 0.05). Grain yield was 6.82–7.36 t/ha in ER and 7.40–7.98 t/ha in LR with a sowing date delay of 0–10 days. In LR, filling rate decreased significantly (by 8.85%, with a 5-day interval) as sowing date delay increased. We detected significant positive correlations between effective accumulated temperature in the post-heading stage and yield (*P* < 0.01) and filling ratio (*P* < 0.01) in LR (Table 5). No significant differences were observed in panicles m^−2^, spikelet panicle^−1^, or grain weight among treatments in ER or LR. Therefore, sufficient effective accumulated temperature (low daily temperature occurred at anthesis with delay ESD, Fig. 1a,b) at anthesis was the key to determining ESD in LR to maintain high grain yield.

**Table 4:** Grain yield in machine-transplanted double-cropped rice grown under different sowing periods in 2015 and 2016.

Season	Cultivar	Sowing date delay (days)	Yield (t/ha)	Panicle m^−2^	Spikelet panicle^−1^	Filling ratio (%)	Grain weight (mg)
2015 ER	ZZ39	+0	6.01	240	110	90.1	25.6
		+5	6.88	264	103	87.8	25.7
		+10	6.84	258	117	89.1	25.6
		+15	5.99	214	135	83.6	24.8
2016 ER	ZZ39	+0	8.61	312	128	81.6	24.3
		+5	7.68	342	114	83.5	25.0
		+10	7.90	291	132	77.1	25.1
		+15	7.39	259	130	80.3	24.4
	LLY268	+0	7.01	387	100	75.4	24.7
		+5	7.36	379	89	82.8	24.9
		+10	6.72	382	105	59.7	24.1
		+15	6.12	381	106	65.8	24.0
		+0	7.36 ± 1.31a	322 ± 74a	113 ± 14a	81.4 ± 7.4a	24.8 ± 0.7a
		+5	7.36 ± 0.40a	337 ± 59a	102 ± 13a	84.3 ± 2.7a	25.2 ± 0.4a
		+10	6.82 ± 0.65a	305 ± 64a	118 ± 14a	73.5 ± 14.8a	24.9 ± 0.8a
		+15	6.56 ± 0.77a	306 ± 86a	122 ± 16a	75.7 ± 9.5a	24.4 ± 0.4a
		LSD0.05	1.60	135	26	18.1	1.1
2015 LR	HY518	+0	6.57	383	91	82.8	25.1
		+5	6.85	367	82	76.1	25.3
		+10	7.22	350	91	79.4	24.6
		+15	5.07	360	101	74.4	24.6
		+20	5.83	398	75	64.5	24.8
2016 LR	HY518	+0	8.81	396	130	78.6	24.8
		+5	8.19	401	125	77.8	23.6
		+10	8.57	446	111	72.4	26.0
		+15	6.90	480	116	59.9	25.4
		+20	5.38	502	137	45.6	22.9
	TY390	+0	7.55	431	133	80.8	20.5
		+5	8.89	413	135	73.1	22.4
		+10	6.42	437	127	61.5	22.8
		+15	5.09	442	145	54.2	20.3
		+20	3.27	561	118	35.5	18.9
		+0	7.64 ± 1.12ab	409 ± 25a	126 ± 23a	80.2 ± 2.1a	22.9 ± 2.6a
		+5	7.98 ± 1.04a	401 ± 24a	123 ± 28a	75.6 ± 2.4ab	23.3 ± 1.5a
		+10	7.40 ± 1.09ab	429 ± 53a	115 ± 18a	68.8 ± 9.0ab	24.4 ± 1.6a
		+15	5.69 ± 1.05bc	429 ± 61a	115 ± 22a	59.5 ± 10.4bc	23.1 ± 2.7a
		+20	4.83 ± 1.37c	512 ± 83a	120 ± 32a	44.0 ± 14.7c	21.4 ± 3.0a
		LSD 0.05	2.07	98	46	16.6	4.3

ER, early rice; LR, late rice. LSD, least significant difference at a significance level of *P* < 0.05.

**Table 5:** Correlations between effective accumulated temperature and other indices.

Yield		Panicle m^−2^		Spikelet Panicle^−1^	Filling ratio	Grain weight	Aboveground biomass	Harvest index
**Early rice (ER)**								
SO–TP	−0.12	−0.08		0.39	−0.11	−0.36	0.16	−0.21
TP–FH	0.19	0.92^***^		−0.45	−0.78^**^	−0.69^*^	0.85^***^	−0.82^**^
FH–MA	−0.33	0.02		0.07	−0.45	−0.35	0.14	−0.46
Total	−0.01	0.75^**^		−0.25	−0.86^***^	−0.80^**^	0.80^**^	−0.92^***^
**Late rice (LR)**								
SO–TP	−0.70^**^		0.02	−0.56^*^	−0.35	0.18	0.59^*^	−0.21
TP–FH	0.47		0.09	0.52^*^	0.19	−0.40	0.50	0.01
FH–MA	0.73^**^		−0.42	0.21	0.68^**^	0.15	0.25	0.54
Total	0.63^*^		−0.17	0.40	0.46	−0.19	0.40	0.27

SO, sowing stage; TP, transplanting stage; FH, full heading stage; MA, mature stage. *, ** and *** denote significant differences at the 0.05, 0.01 and 0.001 probability level, respectively.

## Discussions

### Ensuring elastic sowing date in mechanized large-scale double-cropped rice production

The ER and LR cultivars used in this study are temperature- and light-sensitive, respectively. In a study of two field stations in China during 1981–2000, Tao *et al*.^11^ reported that growth duration was significantly shorter and longer in ER and LR, respectively, as temperature increased. Our data also indicated indirectly that ER growth duration decreased as effective accumulated temperatures increased. In contrast, Zhang *et al*.^12^ demonstrated that planting short-duration cultivars at increasing temperatures exacerbated undesired phenological changes, with the growth duration of the late rice cultivar ‘HY518’ 7–14 days shorter than that of ‘TY390’. In the current study, the reproductive stage of rice sown after a 20-day delay was dramatically prolonged, to 8 days longer than that of the control. Therefore, priority should be given to the ESD of late rice; in long-duration cultivars, reasonable ESD should be set at no later than July 10, especially as the ESD of ER is dependent on that of LR.

### Annual rice grain yield maintenance was dependent on late rice grain yield

In late rice (LR), delayed sowing led to significantly lower grain yield and prolonged growth duration. Similar results have been reported previously, with delayed transplanting reducing spikelet filling and grain yield due to low temperature stress at anthesis in machine-transplanted LR^7^. The number of days with daily mean temperature <22 °C, which is the critical low temperature for anthesis in rice^13^, increased as sowing date was postponed, at 2–10 days in 2015 and 0–7 days in 2016 during the first 10 days after heading. These variation were directly affect the filling rate at the repining stage. Our data indicated that filling rate decreased significantly (by 8.85%, with a 5-day interval) as sowing date delay increased.

In additional, total dry matter of late rice among different sowing dates was averagely 1467~1526 g m^−2^, if HI was normal (about 0.5), theoretical yield was 7.3~7.6 t ha^−1^, our results also suggested that dry matter production was not affected as sowing date was delayed. However, reproductive redundancy increased by 7.72% in LR, with a 5-day interval, following sowing date delay. Possible reason was that the inability to transfer aboveground biomass in the vegetative organs to grain yield in LR due to less effective accumulated temperature or low-temperature stress at anthesis was intensified as sowing date was delayed, although there was no significant correlation between effective accumulated temperature of growth duration after transplanting and aboveground biomass (*P* > 0.05) or HI (*P* > 0.05). Double cropped rice production in China is undergoing an unprecedented period of transition to large-scale mechanization^14^. Precondition of high annual rice yield under the double cropped rice production was high early rice yield and high late rice yield, therefore, reasonable allocation of limited thermo-unit conditions annually was the keys for rice growth and development and even yield. Adjusting sowing date (seedling age was fixed) was for improving allocation of limited thermo-unit conditions under the double cropped rice production. In this study, in view of low temperature stress under late rice season, and to promote high grain yield in mechanized large-scale farming, we examined ESD for LR, and determined that it should be no later than July 10 (a 10-day delay compared with the control), and that ESD for ER should be 10 days earlier than the control, rather than later. Even if reasonable ESD is adopted, the potential threat of meteorological factors, especially high and low temperatures^15,16^, remain a threat to sustainably increasing grain yield. The increase in reproductive redundancy observed in this study indicates that affecting yield remains a risk in this approach. Therefore, our results suggest that greater effort should be made to develop high-yield multi-resistant rice cultivars to meet the development of mechanized large-scale rice farming, whether via conventional breeding, biotechnology, or both^17^. Growth duration of rice cultivars must also be considered^18^; our data demonstrate that thermal sensitive long-duration rice cultivars are appropriate for the early season, and day-neutral and thermal sensitive short-duration cultivars for the late season (e.g. ^6,12^).

## Conclusions

Elastic sowing dates (ESD) with low seeding rate was conductive to grain yield maintenance in mechanized large-scale double-cropped rice production. ESD for LR should be no later than July 10 (a 10-day delay compared with the control), and ESD for ER should be earlier sowing rather than later.

## Materials and Methods

### Experiments designs

**Figure 2:**
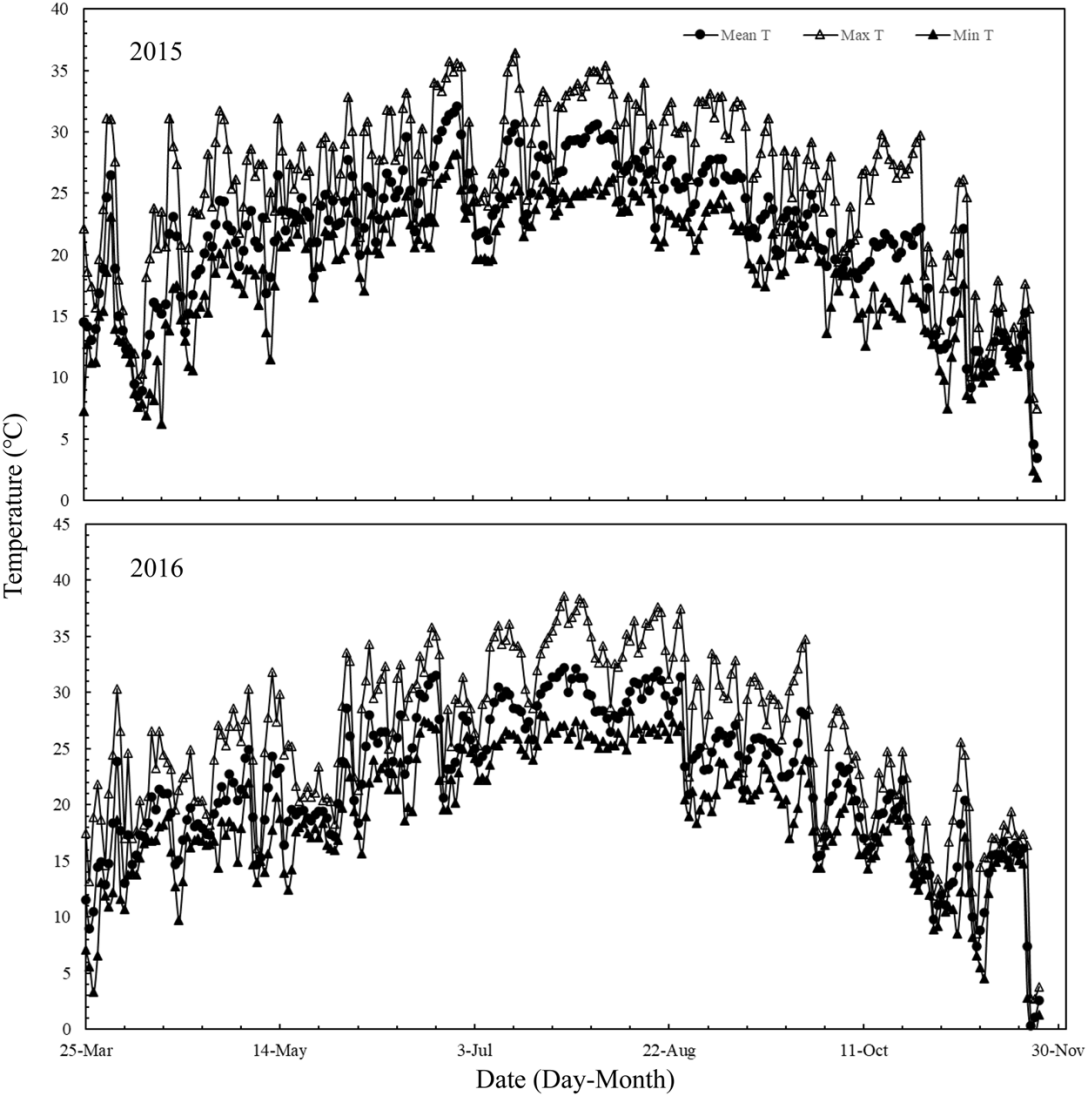
Daily mean temperature (Mean T), maximum temperature (Max T), minimum temperature (Min T) and solar radiation during the rice growing season from seeding to maturity of double rice in Yongan County, Hunan Province, China in 2015–2016.

**Figure 3:**
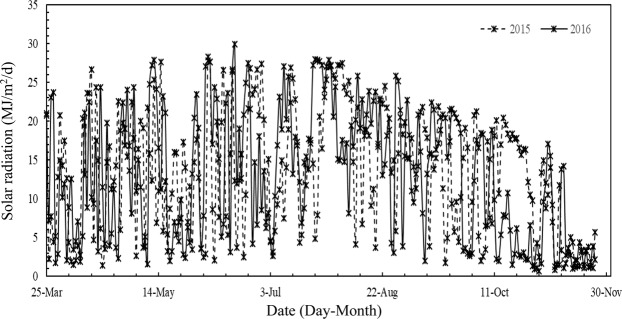
Solar radiation during the rice growing season from seeding to maturity of double rice in Yongan County, Hunan Province, China in 2015–2016.

Field experiments were conducted in Yongan, Hunan Province, China (28°09′N, 113°37′E, 43 m a.s.l.) in the early and late seasons in 2015 and 2016. Maximum and minimum temperatures between March 25 and November 22 were 26.8 °C and 19.3 °C in 2015, and 26.4 °C and 19.3 °C in 2016, respectively (Fig. 2). High temperatures (daily maximum temperature ≥35 °C) occurred more frequently in 2016 than in 2015. Solar radiation between March 25 and November 22 were 13.9MJ/m^2^/d in 2015, and 13.3MJ/m^2^/d in 2016, respectively (Fig. 3). The soil was clayey with a pH of averagely 6.30, organic matter content of averagely 18.4 g kg^−1^, and total nitrogen (N) content of averagely 1.09 g kg^−1^. We performed a soil test using samples collected from the upper 20 cm. The ER cultivars used in this study were Zhongzao 39 (ZZ39, inbred) in 2015 and Lingliangyou268 (LLY268, hybrid) in 2016. The LR cultivars were Hyou 518 (HY518, hybrid) in 2015 and Taiyou390 (TY390, hybrid) in 2016. These four cultivars were selected because they are widely grown by rice farmers in the study region.

Rice planting in a randomized block design was established with different sowing dates and three replicates in a 25-m^2^ plot. We planted 20-day-old ER seedlings on four sowing dates between March 25 (control) and April 9, at 5-day intervals. We planted 15-day-old LR seedlings on five sowing dates between July 1 (control) and July 21, at 5-day intervals.

Seeding was performed on paper using a single-seed printing seeder (HDBZJ-580-A, Hande Co. Ltd.), and sowing was performed using seedling trays (length × width × height = 58 cm × 25 cm × 2 cm) at a rate of 15 g per tray. According to local recommended density of ER and LR, the transplanting density was 28.5 hills per m^2^ for early season (ER, row spacing × planting spacing = 25 cm × 14 cm), and 36.4 hills per m^2^ for late season (LR, 25 cm × 11 cm).

One seedling was transplanted per hill. N content of the soil was 150.0 kg N ha^−1^ for ES and 165.0 kg N ha^−1^ for LS, with 70% of total N at basal dressing and 30% of total N at panicle initiation. Phosphorus (P) and potassium (K) rates were 75.0 kg P_2_O_5_ ha^−1^ and 120.0 kg K_2_O ha^−1^ for ES and 82.5 kg P_2_O_5_ ha^−1^ and 132.0 kg K_2_O ha^−1^ for LS. P was applied initially; K application was split equally between initial application after transplantation and at panicle initiation. The water management strategy was flooding, followed by midseason drainage, re-flooding, moist intermittent irrigation, and drainage. Weeds, insects, and diseases were intensively controlled with chemicals.

### Growth period, tillers, dry matter and yield attribute sampling

Dates of sowing and the full-heading and mature stages were recorded accurately. Excluding the three border plants, 10 hills were labeled in each plot to count tillers at fixed intervals from 5 to 40 days after transplanting; 10 hills were sampled and aboveground biomass was determined in the flowering stage. In the mature stage, yield components were determined for 12 hills, including spikelet panicle^−1^, filling ratio, and grain weight. Finally, grain yield was determined in a selected 5-m^2^ area, and the effective number of panicles per m^2^ was determined for 20 hills.

Nutrition redundancy was calculated from the difference between the maximum number of tillers and the effective number of panicles per m^2^. Reproductive redundancy was calculated from the barren grain number and total grain number. Effective accumulated temperature at the different stages was calculated as the difference between daily average temperature and biological initial temperature (10 °C, Indica rice).

### Statistical analysis

Statistical analyses were performed using analysis of variance (ANOVA) with Statistix 8 software (Analytical Software, Tallahassee, FL, USA). Treatment means and years were compared based on the least significant difference test at a significance level of 0.05.
